# Enhancing satiety and aerobic performance with beer microparticles-based non-alcoholic drinks: exploring dose and duration effects

**DOI:** 10.3389/fnut.2023.1225189

**Published:** 2024-01-03

**Authors:** Fábio Luiz Candido Cahuê, Paola D. D. S. Maia, Luan Ribeiro de Brito, Victor Paulo Ferreira da Silva, Diego Viana Gomes, Anna Paola T. R. Pierucci

**Affiliations:** Basic and Experimental Nutrition Department, Josué de Castro Nutrition Institute, Federal University of Rio de Janeiro, Rio de Janeiro, Brazil

**Keywords:** beer, aerobic training exercise, microparticles, running performance, satiety and food intake

## Abstract

Beer is an alcoholic beverage, rich in carbohydrates, amino acids, vitamins and polyphenols, consumed worldwide as a social drink. There is a large number of beer styles which depends on the ingredients and brewing process. The consumption of beer as a fluid replacement after sport practice is a current discussion in literature. A non-alcoholic pale-ale microparticles-based beverage (PABM) have been previously designed, however, its phenolic profile and ergogenic effect remain unknown. Thus, this study aims to verify the ergogenic potential (increase of running performance) of PAMB in male Wistar rats. Beer microparticles were obtained by spray drying and beverages with different concentrations were prepared in water. Wistar rats were subjected to a training protocol on a treadmill (5 times/week, 60 min/day) and daily intake of PABM (20 mg.kg-1 or 200 mg.kg-1) or water by gavage. Chlorogenic acid was found to be the main component in the phenolic profile (12.28 mg·g-1) of PABM analyzed with high-performance liquid chromatography and mass spectrometry. An increase in the aerobic performance was observed after 4 weeks in the 20 mg.kg-1 group, but the same dose after 8 weeks and a higher dose (200 mg.kg-1) blunted this effect. A higher dose was also related to decrease in food intake. These data suggest that PABM can improve satiety and aerobic performance, but its effect depends on the dose and time of consumption.

## 1 Introduction

Beer is a widely consumed alcoholic fermented beverage that is rich in bioactive compounds, antioxidants, and exhibits anti-inflammatory activity. It consists of a complex mixture of carbohydrates, vitamins, and minerals, offering unique organoleptic properties (1–4).

Furthermore, beer is a sensorially diverse beverage that can be categorized into various styles based on factors such as ingredients, formulation, fermentation type, manufacturing process, alcohol content, color, flavor, and aging. These factors directly influence the composition of polyphenols present in the beer (5).

The phenolic compounds found in beer primarily originate from the chemical transformations of malt and hops during the brewing process. Malt contributes around 70% of the beverage’s polyphenols, while hops contribute approximately 30% (6). Comparatively, ale beers possess higher total phenolic content (TFC) and antioxidant activity (AA) than lagers. This disparity is due to the higher fermentation temperature required for brewing lagers, resulting in increased extraction of phenolics during the process (7).

Metabolomic analysis of different brands of ale and lager has revealed that the principal metabolites responsible for their distinctive characteristics are the polyphenols derived from the varying qualities and quantities of malt and hops used in their formulations. Consequently, beers with stronger profiles tend to exhibit higher overall content of these metabolites (6).

Athletes consume substantial quantities of beer, not only as a social drink (1) but also as a fluid replacement after practicing sports (2). The potential of beer intake for rehydration has been evaluated before or after exercise and at different stages of dehydration (3, 4, 8, 9). However, the intake of beer during the exercise recovery phase is questionable because of the required alcohol content in the volume for adequate rehydration. Alcohol ingestion can suppress the anabolic response in skeletal muscles and impair recovery and adaptation to training and/or subsequent performance (5).

Recently, we designed an innovative non-alcoholic beer-based functional beverage with excellent yield process of spray-drying ranged from 70.0% to 74.7%, high solubility (>90%), low a_w_ values ranging from 0.206 to 0.282, good particle size distribution were expressed as d [0.5], ranging from 0.58 to 0.84 μm, antioxidant activity evaluated by FRAP, TEAC, and total phenolic compounds showed high values and presented percentage retentions ranging from 80% to 98% (6). Furthermore, the non-alcoholic beer-based functional beverage showed a slightly more spherical conformation, with little invaginations and roughness. This beer powders developed showed 3.07 ± 0.02% of ash, 3.9 ± 0.01% of moisture, 85.3 ± 0.04 g of carbohydrate, 7.4 ± 0.02 g of protein, lipids were not detected and did not contribute to the energy value, and 370.8 ± 0.06 kcal of energy value. Calcium, sodium, potassium, and magnesium were the minerals identified and quantified in the beer powders, with values of 448 ± 0.11, 2,000 ± 0.05, 10,227 ± 0.03 and 1,880 ± 0.02 mg·kg-1, respectively.

In addition, non-alcoholic beer-based functional beverage received relatively high mean scores for all sensory attributes, varying from 5.6 to 7.1 in the nine point hedonic scale, which corresponded from “I slightly liked” to “I liked” and the overall acceptability for received average scores corresponding to “really liked,” indicating a high product acceptance (7). However, the long-term effects of its consumption are still unknown. Thus, this study aimed to evaluate whether a beverage containing beer microparticles has ergogenic effects (described in this study as running performance improvement) in association with long-term exercise protocols in Wistar rats. Furthermore, the study aims to assess the impact of the consumption of beer microparticles-based beverage on eating behavior.

## 2 Materials and methods

### 2.1 Preparation and analysis of pale ale beer microparticles

#### 2.1.1 Pale ale craft beer microparticles preparation

Pale ale craft beer was developed at the Institute of Microbiology, Professor Paulo de Goes/UFRJ, as described before (6). The pale ale beer microparticles (PABM) were prepared by spray-drying using a Mini Spray Dryer Büchi model B-290 (Büchi Laboratoriums Technik, Flawil, Switzerland) in triplicate, and the powdered were stored at −80°C. The operating conditions were a sample feed flow of 0.36 L.h^−1^, an aspiration rate of 32 m^3^.h^−1^, a compressor air pressure of 0.03 MPa, an inlet air temperature of 180°C, and a 0.7 mm diameter nozzle.

#### 2.1.2 Microparticle morphology

The morphology of microparticle was evaluated by scanning electron microscopy (SEM) through the model JSM 5310 (JEOL^®^, Tokyo, Japan) as described before (10).

#### 2.1.3 Proximal composition and physicochemical analysis

The protein, lipid, moisture and ash compositions were determined in samples microparticles according to the Association of Official Analytical Chemists (11). The total carbohydrates were estimated by subtracting the sum of moisture, ash, protein, and lipids from 100%. The general factor system was used in the proximate chemical composition to calculate the energy values. The minerals such as sodium (Na), magnesium (Mg), calcium (Ca) and potassium (K) were identified and quantified according to the method described previously (12) using spectrometry of atomic absorption (Shimadzu AA-6800, Tokyo, Japan). The preparation consists of weighing 3 g of each sample into porcelain crucibles and placing them in a muffle furnace until the organic material is completely burnt off, enough time for the material to turn white. In the wet ash determination method, concentrated sulfuric acid is used at high temperatures to cause the organic matter to decompose and each mineral to be analyzed individually. The quantification of minerals was carried out by interpolation using an analytical curve with standard solutions for calibration. The calibration solutions were prepared from the dilution of SpecSol standard stock solution with a concentration of 1,000 or 10,000 mg L-1 (Quimlab Química & Metrologia^®^, Jardim California, Jacareí, São Paulo, Brazil) until the desired concentrations were obtained, using matrix resemblance.

#### 2.1.4 Antioxidant activity and total phenolic content

About 10 mg of pale ale craft beer microparticles were homogenized in 1.0 mL distilled water and stored in a water bath at 90°C for 3 h. After cooling, methanol (1.0 mL) was added, and centrifuged for 5 min (1,500 × g). The AA was determined using the ferric-reducing ability of plasma (FRAP) (13) and Trolox equivalent antioxidant capacity (TEAC) assays (14) in triplicate. The FRAP and TEAC results are expressed as μmol Fe^2+^·g^−1^ and μmol Trolox·g^−1^ on a dry weight basis (dwb), respectively. The Folin–Ciocalteu assay was used to determine TPC in the sample’s beer, as described by Singleton et al. (15) and modified before (16). The results are expressed as mg of gallic acid equivalents (GAE)·g^−1^ dwb.

#### 2.1.5 Production of phenolic extract

The extraction of soluble phenolic compounds (PCs) from samples of pale ale craft beer was according to a customized methodology described before (17). Cold ethanol in a deionized and distilled water solution (80,20, v/v), stirred for 10 min, and then centrifuged at 2500 × g for 5 min at 10°C was used to extracted soluble PCs. A rotary evaporator at 130 rpm were used to remove the solvents and the dry residues were reconstituted in water. The extract was stored at-80°C until it was provided to the mice.

#### 2.1.6 Phenolic compound contents

The PC content analysis of pale ale craft beer microparticles was performed according to the adaptations made previously (18) using soluble and conjugate phenolic extraction methods. Conjugated PCs were extracted via alkaline and acid hydrolysis. All extracts were filtered through 0.45 μm cellulose ester membranes (Merck Millipore Co., Darmstadt, Germany) prior to high-performance liquid chromatography (HPLC). The HPLC system was equipped with a 5 μm C18 guard column (10 × 3.0 mm, I.D., Ascentis^®^), 5 μm reverse-phase C18 column (250 × 4.6 mm, I.D., Ascentis^®^, Los Angeles, CA, United States) and SPD-M30A (Shimadzu, Kyoto, Japan) diode-array detector (DAD). The DAD wavelength was monitored from 190 to 370 nm. The temperature of column was set at 40°C and the volume of injection was 20 μL. The mobile phase (1.0 mL·min^−1^) was 0.3% formic acid (in H_2_O-DD), methanol (100%), and acetonitrile (100%) with gradient elution. The quantification of PC was performed using a calibration curve (1–50 ppm) with a minimum of five standards concentrations.

### 2.2 Exercise performance protocol

#### 2.2.1 Animals

Adult male Wistar rats (8 weeks old, weight 284 ± 28 g) were used in the experiments because of the high similarity of response to exercise training when compared to humans (19). Wistar rats were housed in a temperature-controlled (22 ± 2°C) environment with a light/dark cycle. The animals had access to commercial rat chow (NOVA LAB; Rio de Janeiro, Brazil) and tap water. The study was approved by the Ethics Committee for Ethics in Animal Use (CEUA) in Scientific Experimentation of the Health Sciences Center of the Federal University of Rio de Janeiro, registered with the National Council for Animal Experimentation Control (CONCEA) under case number 01200.001568/2013-87.

#### 2.2.2 Study design

The animals were separated into three groups: trained with tap water (Control, *n* = 5), trained with 20 mg.kg^−1^ body weight of PABM (PABM20, *n* = 5), and trained with 200 mg.kg^−1^ body weight of PABM (PABM200, *n* = 5). The beverages were prepared by diluting PABM with tap water and homogenization with an Ultra-Turrax^®^ (IKA) at 3000 rpm for 5 min immediately before administration to the animals. Animals received oral gavage daily, in the morning, during all periods of the experiment. The dosages of the beer microparticles tested in this study were based on previous studies, given the potential delivery of phenolic compounds (20–23).

#### 2.2.3 Exercise protocol

**Figure 1:**
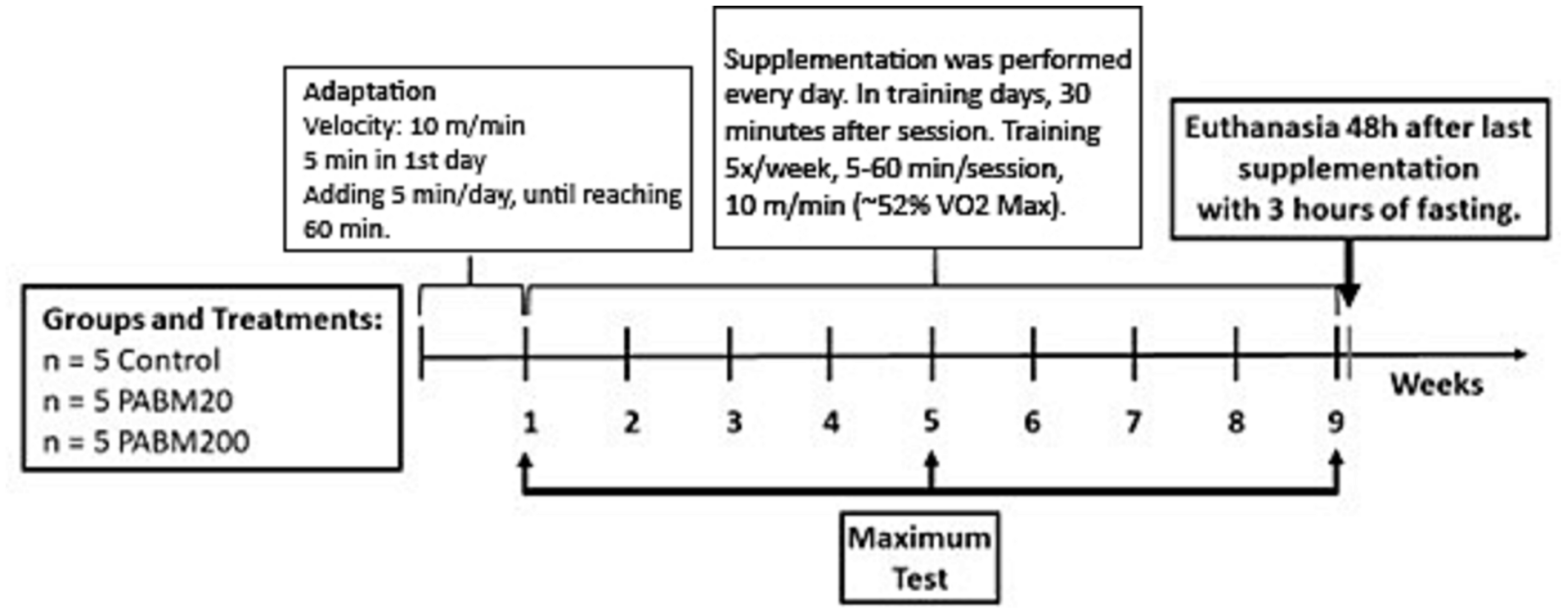
Experimental design of the chronic protocol for supplementation with the pale ale beer microparticles (PABM). Control group (trained and treated with water); Group PABM20 (trained and treated with 20 mg/kg of beer microparticles), and Group PABM200 (trained and treated with 200 mg/kg of beer microparticles).

In accordance with Figure 1, the experimental animals underwent treadmill exercise at an initial inclination of 15°, running at a constant velocity of 10 m/min (~52% VO2_Max_) (24) for 5 min on the first day. Subsequently, the daily exercise duration was incrementally augmented by 5 min per day until a final target of 60 min per day was attained, and this duration was sustained throughout the entire experimental protocol. The exercise training regimen spanned a total of 8 weeks, with the animals exercising on the treadmill for 60 min, five times per week, in strict adherence to the prescribed recommendations set forth in the Guidelines for Animal Exercise and Training Protocols (25).

#### 2.2.4 Maximum aerobic capacity test

To determine the maximum aerobic capacity, the animals started the protocol on a treadmill at a speed of 10 m/min, and every 2 min of running the speed was increased by 2 m/min until the animals reached exhaustion, which consisted of standing still on the shock grid for more than 5 s. The tests were performed before starting physical training, and the animals were tested again after four weeks, and the last test was performed after the eighth week of physical training. The physical performance parameters analyzed were time, total distance, and maximum speed (22).

#### 2.2.5 Weight gain and food intake assessment

For the chronic protocol, the body masses of the animals were evaluated on a high-precision digital scale (Digimed^®^) at an interval of every two days. The difference between the values (final and initial) was considered to be the weight gain during the period. For the assessment of food intake, 300 g of feed (standard chow – Biobase^®^, Brazil), was placed in each box every two days and weighed until there was more food to be replaced.

#### 2.2.6 Blood sampling and analyses

Immediately after each maximum aerobic exercise test using both protocols, blood samples were collected. Aspartate Aminotransferase (ALT), Alanine Aminotransferase (AST,) and Creatine Kinase (CK) levels were analyzed using commercial kits (BioClin, Goiás, Goiânia, Brazil) according to the manufacturer’s instructions. All measurements were performed in duplicate and a biocontrol with pathological and normal parameters was used to monitor the accuracy and precision of the analytical testing using manual and automated methodologies. The total antioxidant capacity was measured using the FRAP method (13).

### 2.3 Statistical analysis

*A priori* power analysis with an effect size of 0.90 for performance gain in the time-to-exhaustion test, an α level of 0.05, and β level of 0.20 (80% power) indicated a requirement for five participants per group. Shapiro–Wilk normality test was used to confirm normal distribution of data. One-or two-way analysis of variance (ANOVA) was used to compare the means between the groups. When appropriate, a two-stage linear step-up procedure of the Benjamini, Krieger, and Yekutieli method for multiple comparison testing was performed. Eta-squared or partial eta-squared was used to evaluate effect size. The values of η^2^ or partial η^2^ for small, medium and large effect was 0.01, 0.06, and 0.16, respectively (26). All statistical analyses were performed using GraphPad Prism software (version 8.0; La Jolla, CA, United States). The data are presented as means ± the standard deviation (SD). Comparisons were considered statistically significant when *p* < = 0.05 and *q* (false discovery rate) < = 0.1.

## 3 Results

### 3.1 Microparticle morphology

**Figure 2:**
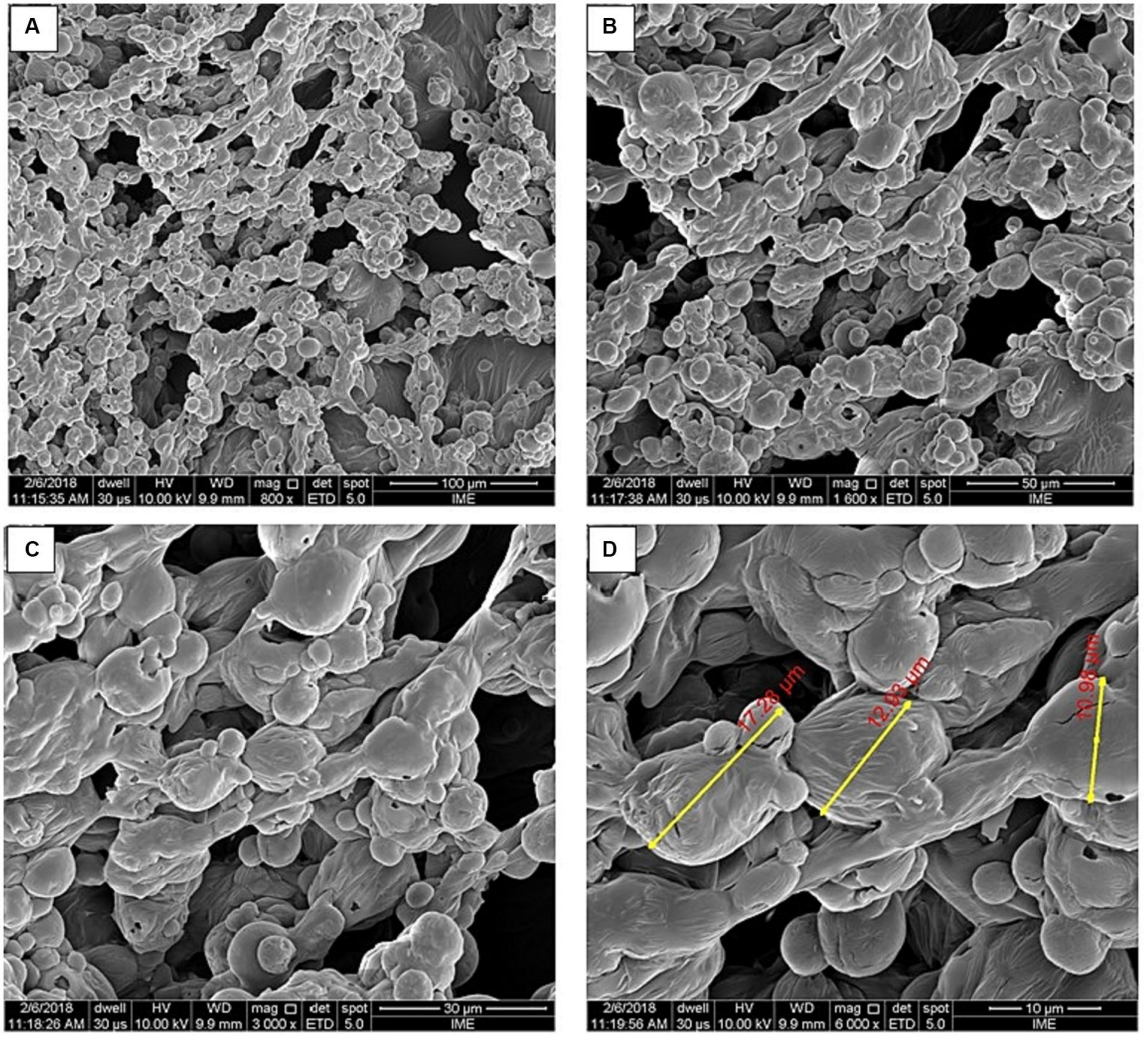
Representative scanning electron microscopy micrographs of microparticles obtained from spray-drying pale ale craft beer. **(A)** 100 μm size bar, 800x magnification; **(B)** 50 μm size bar, 1,600x magnification; **(C)** 30 μm size bar, 3,000x magnification; and **(D)** 10 μm size bar, 6,000x magnification.

The morphological characterization of the microparticles of the pale ale craft beer by SEM showed a slightly more spherical conformation without invaginations and roughness (Figure 2).

### 3.2 Proximal composition and physicochemical analysis

Table 1 presents the proximal composition and physicochemical analysis of the PABM. Moisture (3.6%), protein (7.8 g), carbohydrate (86.3 g), and ash (3.0%) contents were observed in the beer microparticles. Lipids were not detected in the beer powders and did not contribute to the energy value of the PABM. Ca, Na, K, and Mg were identified as the main minerals (Table 1). The beer powder exhibited a solubility of 92.35 ± 0.52% and water activity (a_w_) of 0.27 ± 0.01. FRAP and TEAC assays yielded values 8873.10 ± 7.92 μmol Fe^2+^·g^−1^ and 1449.15 ± 4.50 Trolox·g^−1^, respectively, while TPC was evaluated using the Folin–Ciocalteu extraction method and was 910.30 ± 1.91 mg GAE·g^−1^ (Table 1).

**Table 1:** Proximal composition and physicochemical analysis of pale ale beer microparticles (PABM).

Variables	PABM (Mean ± SD)
Proximate composition (g·100 g^−1^ PABM)	
Moisture (%)	3.6 ± 0.01
Energy (kilocalories)	376.4 ± 0.06
Protein (g)	7.8 ± 0.02
Carbohydrates (g)	86.3 ± 0.04
Lipids (g)	ND
Ashes (%)	3.0 ± 0.02
Physicochemical characteristics	
Solubility (%)	92.3 ± 0.52
a_w_	0.27 ± 0.01
TPC (mg GAE·g^−1^)	910.30 ± 1.91
FRAP (μmol Fe^2+^·g^−1^)	8873.10 ± 7.92
TEAC (μmol Trolox·g^−1^)	1449.15 ± 4.50
Minerals (mg·kg^−1^ PABM)	
Calcium (mg·kg^−1^)	498 ± 0.10
Magnesium (mg·kg^−1^)	140.1 ± 0.05
Potassium (mg·kg^−1^)	10,227 ± 0.03
Sodium (mg·kg^−1^)	1900 ± 0.05

The values are means ± SD. Different letters in the same column indicate statistically significant differences among samples (*p* < 0.05). a_w_, water activity; TPC, total phenolic compounds; GAE, gallic acid equivalent; FRAP, ferric reducing antioxidant power; TEAC, trolox equivalent antioxidant capacity.

### 3.3 Identification and quantification of phenolic compounds

The total PC content in 20 mg and 200 mg in the PABM was 2.463 ± 0.379 and 24.632 ± 3.79 mg.g^−1^ of fwb, respectively (Table 2). Ten different PCs were identified and quantified in beer phenolic extract, such as di-OH-benzoic, gallic, p-coumaric, m-coumaric, vanillic, rosmarinic, ferulic, syringic, chlorogenic and 4-OH-phenylacetic acids. Considering only the soluble phenolic extract, 4-OH-phenylacetic, gallic, di-OH-benzoic, chlorogenic and vanillic acids were the most abundant detected in the beer phenolic extract (BPE). Gallic, rosmarinic, vanillic, and 4-OH-phenylacetic acids were found in both the soluble and insoluble BPE.

**Table 2:** Phenolic compounds contents in 20 mg and 200 mg of beer phenolic extract (BPE).

	BPE (mg·20 mg^−1^ fwb)	BPE (mg·200 mg^−1^ fwb)
Chlorogenic acid	0.245 ± 0.001^a^	2.456 ± 0.216^a^
Di-OH-benzoic acid	0.227 ± 0.016^a,b^	2.278 ± 0.164^a,b^
Ferulic acid	0.162 ± 0.024^c,d^	1.618 ± 0.241^c,d^
Gallic acid	0.221 ± 0.011^a,b^	2.216 ± 0.114^a,b^
*m*-Coumaric	0.186 ± 0.022^c,d^	1.864 ± 0.218^c,d^
*p*-Coumaric	0.102 ± 0.047^e^	1.024 ± 0.479^e^
Rosmarinic acid	0.132 ± 0.011^e^	1.322 ± 0.116^e^
Syringic acid	0.147 ± 0.042^d,e^	1.474 ± 0.424^d,e^
Vanillic acid	0.236 ± 0.021^a,b^	2.364 ± 0.211^a,b^
4-OH-phenylacetic acid	0.184 ± 0.038^b,c,d^	1.842 ± 0.378^b,c,d^
Gallic acid	0.152 ± 0.002^a^	1.518 ± 0.022^a^
Rosmarinic acid	0.148 ± 0.031^a^	1.488 ± 0.302^a^
Vanillic acid	0.166 ± 0.027^a^	1.664 ± 0.278^a^
4-OH-phenylacetic acid	0.151 ± 0.006^a^	1.504 ± 0.058^a^
**Total**	**2.463 ± 0.379**	**24.632 ± 3.79**

The values are means ± SD for triplicate analysis. Different letters within the same column for each beer product indicate statistically significant differences between phenolic compounds at a significance level of *p* < 0.05. * Denotes the difference from BPE at a significance level of *p* < 0.01.

### 3.4 Aerobic exercise performance

**Figure 3:**
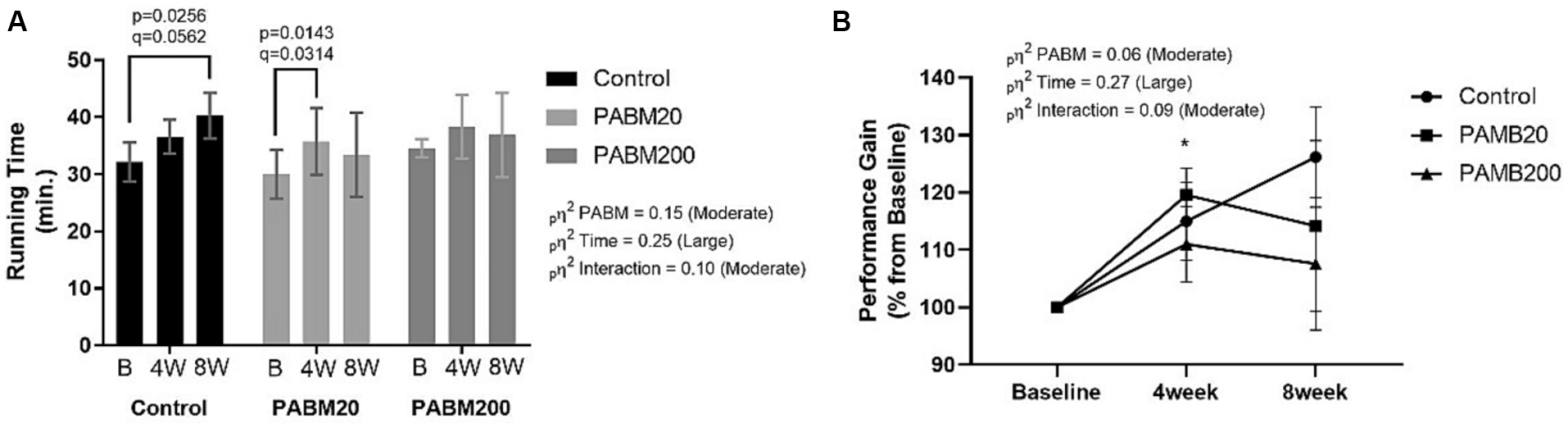
Aerobic exercise performance during and after exercise training protocol. **(A)** Maximum running time in aerobic test until exhaustion. **(B)** Percentage of performance gain in 4 and 8 weeks of the exercise training protocol. Two-way ANOVA with the two-stage linear step-up procedure of the Benjamini, Krieger, and Yekutieli multiple comparisons test was used to evaluate statistical differences between groups. *p < 0.05 and *q* < 0.1 PABM20 vs. Control group.

To evaluate the effect of long-term consumption of beer microparticles on aerobic exercise performance, the maximum exercise test was performed before, after 4 weeks (middle) and after 8 weeks of training (final). As shown in Figure 3, for the control group, the running time was significantly higher after 8 weeks than at baseline (*p* = 0.0256, *q* = 0.0562). In the PABM20 group, the aerobic performance significantly increased after 4 weeks (*p* = 0.0143, *q* = 0.0314), with no difference after 8 weeks. In the PABM200 group, there was no significant increase in aerobic performance after 4 and 8 weeks of exercise training. When the percentage of performance gain was analyzed, only the PABM20 group showed a significant increase after 4 weeks, with no difference after 8 weeks of training. Effect size showed moderate impact of PABM and interaction with time on running performance.

### 3.5 Cell injury biomarkers and total antioxidant capacity

To verify the impact of PABM consumption on antioxidant capacity and injury biomarkers, we analyzed CK, ALT, AST, and FRAP plasma levels after the experimental period. No statistical differences were found between the groups in any of the analyses, but effect size of PABM was moderate in ALT (η^2^ = 0.15) and FRAP (η^2^ = 0.11) and large in AST (η^2^ = 0.16). The results are shown in Supplementary Table S1.

### 3.6 Food intake during chronic treatment

**Figure 4:**
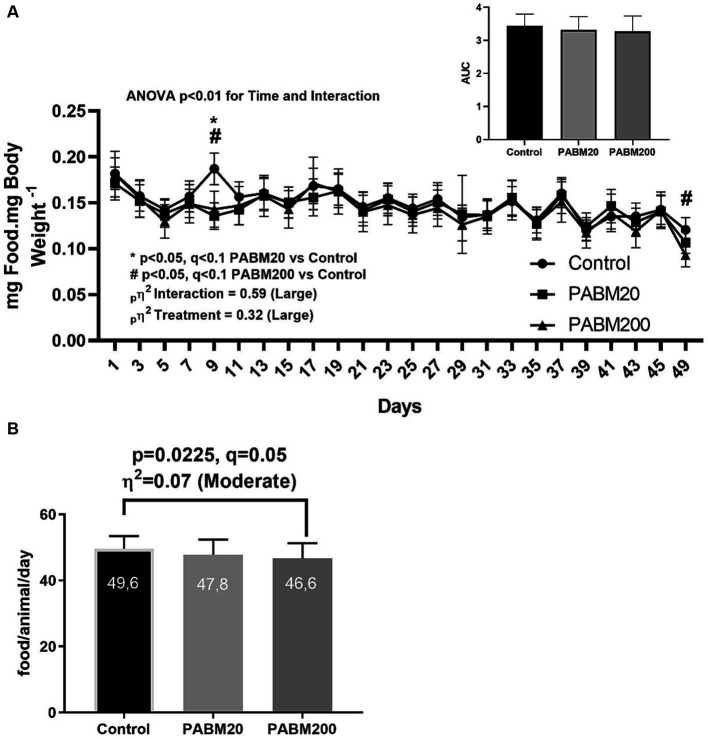
Food intake normalized by body weight gain each 2 days during experimental timeline **(A)**. Food Intake mean considering all experimental period in each group **(B)**. Two-way ANOVA with the two-stage linear step-up procedure of the Benjamini, Krieger, and Yekutieli multiple comparisons test was used to evaluate statistical differences between groups.

Food intake and weight gain were also assessed during the experimental period. Although no differences in weight gain were detected between the groups during the treatment, a significant decrease in food intake was detected with reconstituted beer powder treatment. As shown in Figure 4A, there was a lower food intake, normalized to weight gain, in the experimental groups on day 9 and only in PABM200 on day 49, with a large effect size of PABM (_p_η^2^ = 0.32) and interaction (_p_η^2^ = 0.59). When we analyzed only the mean food intake during the treatment period Figure 4B, a significant decrease was observed in the PABM200 group (*p* = 0.0225, *q* = 0.05), with a moderate effect size of treatment (η^2^ = 0.07).

## 4 Discussion

This study evaluated the ergogenic and satiety effects of pale PABM-based beverages in association with long-term exercise protocols on Wistar rats. Although pale ale craft beer is rich in phenolic compounds (PC), which exhibit antioxidant activity, there is a lack of consensus on the relationship between antioxidant supplementation exercise adaptation (27, 28). Therefore, it is difficult to compare these studies because they used different experimental protocols regarding exercise training intensity and duration, antioxidant supplementation (doses and types of antioxidants), and the molecular parameters analyzed. Gomez-Cabrera et al. (28) found that the endurance time was increased (∼190%) in rats after 6 weeks of training, which was blunted when these animals were supplemented with vitamin C. On the other hand, Higashida et al. (27) found that very large doses of antioxidant vitamins did not prevent the exercise-induced adaptive response. Generally, the evidence tends to show that antioxidant supplementation during training exercise does not result in a beneficial effect (29), however, no scientific study to date has evaluated the antioxidant effect of beer on the physical performance.

Liquid formulations transformation into powders products through spray drying process can provide greater benefits than the products obtained from natural resources. Furthermore, this process has several advantages for the transport of beer, such as volume and mass reduced by removing water and alcohol, stabilization and concentrating nutrients and bioactive compounds. Components that are sensitive to oxidation and heat maintained their stability, similar to the sensory characteristics of color and flavor.

In the present study, PABM presented moisture, proteins, ashes, carbohydrates, solubility, a_w_, TPC, FRAP, TEAC, and mineral content, based on Maia et al. (6) who also characterized beer powder. According to Neto et al. (30), PC in beer help in maintaining the endothelial functional integrity by reducing the formation of superoxide anion, thereby preventing the oxidation of low-density lipoproteins and modulating macrophage activity in endothelium. In subsequent studies, a reduction in systolic blood pressure, homocysteine content, and several inflammatory markers were observed after the consumption of non-alcoholic beer (31, 32).

The microstructure of dry particles in spray-drying can be altered by a variety of factors, such as composition and encapsulating matrix properties, the core to wall material ratio or encapsulating matrix absence, as well as drying and storage conditions (33). The presence of invaginations in the microparticles is due to the viscoelastic properties of the wall material and use of high drying rates, thus favoring the unequal shrinkage of the particles in the initial stages of the process (34). The invaginations on the microparticles adversely affected the powder properties. Microparticles with invaginated or roughened surfaces may exhibit a greater release of the encapsulated core, due to the larger surface contact with the external medium (7, 34). Specifically, the solubility and uptake of water by particles may be enhanced due to the higher surface area than for the smooth ones, which might interfere with the core stability during storage (35).

A low a_w_ value of 0.27 ± 0.01 was observed for microparticles. The inhibition of microbial growth and enzymatic and non-enzymatic degradation occurs within this a_w_ range (36), suggesting a protection against product deterioration.

The potential use of BM derived from the handcrafted pale ale craft beer is based on the concentration of polyphenols. Bertuzzi et al. (37) showed that small-scale beers had a higher TPC and AA values than the large-scale beers. Usually, craft beers (small scale) are unfiltered and unpasteurized, which are the processes that decrease TPC, but there is also a high prevalence of red and wheat beers among them, which are produced with naturally richer raw materials (38). Regardless of the beer type, more than 40 PC have been identified in beer, including simple phenolic acids, hydroxycinnamoylquinic acids, flavanols, flavonols, flavones, alkylmethoxyphenols, alpha-and iso-alpha-acids, hydroxyphenylacetic acids, and prenylflavonoids (39).

Despite the physiological benefits of beer, its alcoholic content is considered a limiting factor for its use as a functional drink. Alcohol-free and alcohol-reduced beverages have increased technological and economic concerns that influence the costs and benefits of the process. Various physical and biological methods have been proposed and applied for the industrial production of alcohol-free and low-alcohol beer, including fermentation-free brewing, dilution procedures, alcohol removal/dealcoholizing, and restricted alcohol fermentation (40, 41). However, the available methods result in significant changes in the nutritional and polyphenol composition of the final product. Low-or non-alcoholic beers have lower TPC and consequently reduced AA compared to the regular beers (42, 43). Therefore, considering the high demand for food products that are nutritious, functional, attractive, with clean labels, and ready for consumption (44), alternative processes could be useful for developing beer-derived polyphenols and nutrient-rich ingredients for sports drinks.

In general, athletes use dietary supplements to promote recovery from training, obtain health benefits, treat illnesses, compensate for a poor diet, and/or improve performance. Nutritionists need to combine the timing, optimal dose, and intake duration of dietary supplements with sports performance (45). In this study it was observed that 20 mg of PABM was better than 200 mg for up to 4 weeks to improve running performance, as supplementation with 20 mg for 8 weeks became ineffective and with 200 mg attenuated the gain during the whole experimental period. Furthermore, it appears that the absence of supplementation (control) presents a greater benefit in performance, while the different dosages inhibit the adaptation promoted by physical training.

There have been a few studies to date on beer polyphenols and their effects on physical performance; however, the literature can explain the effects of polyphenols associated with exercise training. Polyphenols, individually or as a mixture, can improve energy metabolism, oxidative stress, and cell damage, enhancing post-workout recovery (46). Oh et al. (47) administered *Ecklonia cava* polyphenol 30 min before aerobic exhaustion testing and found an increase of 2.39 min in the time of exhaustion and 6.5% in the mean VO_2_ max compared to the placebo group. Our data showed no difference in exercise performance until exhaustion after a single dose of reconstituted beer extract (RBE); however, the group that consumed 20 mg PABM had an early response in endurance performance, showing a higher gain in the first 4 weeks compared to the control group. To the best of our knowledge, this is the first demonstration of the potential of beer microparticles-containing beverages to increase performance in aerobic exercise until exhaustion.

A factor that may be related to the lack of response after 8 weeks of exercise training is the attenuation of mitochondrial biogenesis. Long-term ingestion of antioxidant compounds can decrease redox signaling, which is important for PGC1-alpha activation, a key protein in the mitochondrial biogenesis pathway (29). Although there was moderate effect size of PABM in AST and FRAP and large effect size in ALT, there was no effect of PABM consumption on antioxidant capacity and cell damage biomarkers in our experimental design after 8 weeks of treatment. A possible reason for this result could be the low dose used in our study. In most of the studies that involve supplementation with polyphenol mixture extracts, the dose used was approximately 1,000–2000 mg (20–23), whereas 20 and 200 mg were used in our model. However, the effect of PABM consumption on energy metabolism can explain the early response in the time-to-exhaustion performance in the 20 mg PABM group. Allgrove et al. (48) showed the effect of regular dark chocolate consumption (2 weeks) on free fatty acid mobilization without affecting plasma glucose, insulin, glucagon, and cortisol, suggesting a direct effect of chocolate polyphenols on lipid metabolism.

The impact of micronutrients contained in PABM on running performance can be also explained by a high concentration of potassium. The higher dose (200 mg.kg^−1^) contains ~57% of a rat daily need (49). High-intense exercise can elevate plasma potassium concentration and this elevation can be associated with fatigue due to increase in interstitial potassium, reducing the potential of muscle contraction (50). The protocol used to test running performance increases velocity each 2 min and, in higher velocities, the amount of plasma potassium in PABM200 group could trigger muscular fatigue, reducing performance. Other studies can confirm this mechanism analyzing potassium concentration before and after exercise during chronic PABM consumption.

Another effect of PABM consumption detected in our experiment was a decrease in food intake, with a dose-dependent effect, without a decrease in weight gain. The group that received a higher concentration of RBE exhibited a decreased food consumption. This suggested that PABM consumption could modulate food intake and energy metabolism. These effects were compatible with the phenolic profiles found in the beer extract samples. Chlorogenic, gallic, and ferulic acids, which were also found in our samples and other natural product-derived beverages such as mate (*Ilex paraguariensis*) and coffee, have already been shown to exert reduction in food behavior (51). Song et al. (52) reported an increase in the expression of cholecystokinin (CCK), a primary satiety-signaling hormone, in Caco-2 cells after treatment with chlorogenic acid. In this study, we observed an increase in CCK and glucagon-like peptide 1 (GLP-1), another satiety hormone, in the same cell line after treatment with ferulic acid. Thus, the hypothesis that the consumption of non-alcoholic beer extract can reduce food intake can be partially explained by the phenolic profile found in our samples.

Another molecule related to this effect is 8-prenylnaringenin, a polyphenol found in beer. This compound showed antidiabetic effects, with enhanced expression of AS160, increased activity of AMPK in the liver and skeletal muscle, and reduced fatty acid synthesis after 20 weeks of treatment in mice (53). Naringenin, a precursor of 8-prenylnaringenin, induces an increase in the expression of the anorexigenic hormone cholecystokinin *in vitro* (54). The increase in GLUT4 translocation pathway activity and the possible anorexigenic effect of 8-prenylnaringenin and its precursor naringenin can partially explain the reduction in food intake without changes in body weight gain during the experimental period.

A few limitations of this study are: (1) some specific compounds found in beer microparticles that could promote this effect were not identified, which prevented us from confirming whether the effects were caused by a single compound or a synergistic effect of a pool of polyphenols, (2) the biochemical analysis was performed only on the plasma and not on skeletal muscle samples, and (3) the polyphenol profile was determined by targeting the main compounds found in beer, other natural products, and their derivatives. Specific compounds such as 8-prenylnaringenin were not targeted in our experiments.

In summary, our study reveals that prolonged utilization of non-alcoholic PABM-based beverages led to a notable enhancement in exercise performance for up to 4 weeks, yet this effect was mitigated by an extended 8-week regimen, displaying a clear dose-dependent pattern. Intriguingly, the ingestion of beer microparticles corresponded to a reduction in food intake, all while maintaining body weight. To comprehensively elucidate the intricate interplay underlying the augmentation of exercise performance and regulation of food consumption, a deeper exploration into the molecular mechanisms is necessary.

## Data availability statement

The original contributions presented in the study are included in the article/Supplementary material, further inquiries can be directed to the corresponding author.

## Ethics statement

The animal study was approved by Ethics Committee for Ethics in Animal Use (CEUA) in Scientific Experimentation of the Health Sciences Center of the Federal University of Rio de Janeiro. The study was conducted in accordance with the local legislation and institutional requirements.

## Author contributions

FC: formal analysis, investigation, writing – original draft, and visualization. LB: conceptualization, methodology, investigation, and writing – review & editing. PM, VS, DG: investigation, writing – review & editing, and visualization. AP: conceptualization, methodology, resources, writing – original draft, supervision, project administration, and funding acquisition. All authors contributed to the article and approved the submitted version.
