# Supplementary material for: Enhancing satiety and aerobic performance with beer microparticles-based non-alcoholic drinks: exploring dose and duration effects
Source: Front Nutr. 2024 Jan 3;10:1225189. doi: 10.3389/fnut.2023.1225189 (PMC10791988; doi:10.3389/fnut.2023.1225189)
Supplement: Supplementary file 1 [file Table_1.docx]

**Supplementary Table 1** – Blood biomarkers of cell injury and total antioxidant capacity after 8 weeks of training and BM consumption. The data are presented as means ± SEM. No significant differences were found.

|  | **Control** | **PABM20** | **PABM200** |  |  | **η^2^** |  |
| --- | --- | --- | --- | --- | --- | --- | --- |
| **CK (U.L^-1^)** | 1587 ± 118.8 | 1381 ± 197.8 | 1542 ± 243.7 |  |  | 0.05 (Small) |  |
| **AST (U.L^-1^)** | 0.33 ± 0.008 | 0.31 ± 0.002 | 0.32 ± 0.01 |  |  | 0.15 (Large) |  |
| **ALT (U.L^-1^)** | 0.23 ± 0.003 | 0.23 ± 0.004 | 0.24 ± 0.005 |  |  | 0.16 (Moderate) |  |
| **TAC (µMol Fe^2+^.mL^-1^)** | 24.8 ± 2.1 | 23.3 ± 1.9 | 21.7 ± 1.2 |  |  | 0.11 (Moderate) |  |
